# Rapid point-of-care detection of SARS-CoV-2 using reverse transcription loop-mediated isothermal amplification (RT-LAMP)

**DOI:** 10.1186/s12985-020-01435-6

**Published:** 2020-10-21

**Authors:** Lena Mautner, Christin-Kirsty Baillie, Heike Marie Herold, Wolfram Volkwein, Patrick Guertler, Ute Eberle, Nikolaus Ackermann, Andreas Sing, Melanie Pavlovic, Ottmar Goerlich, Ulrich Busch, Lars Wassill, Ingrid Huber, Armin Baiker

**Affiliations:** Bavarian Health and Food Safety Authority, Veterinaerstrasse 2, 85764 Oberschleißheim, Germany

**Keywords:** RT-LAMP, Point-of-care testing, SARS-CoV-2, COVID-19, Rapid testing, No RNA extraction, ORF8, Gene N

## Abstract

**Background:**

Fast, reliable and easy to handle methods are required to facilitate urgently needed point-of-care testing (POCT) in the current coronavirus pandemic. Life-threatening severe acute respiratory syndrome coronavirus 2 (SARS-CoV-2) has rapidly spread all over the world, infecting more than 33,500,000 people and killing over 1 million of them as of October 2020. Infected individuals without any symptoms might still transfer the virus to others underlining the extraordinary transmissibility of this new coronavirus. In order to identify early infections effectively, treat patients on time and control disease spreading, rapid, accurate and onsite testing methods are urgently required.

**Results:**

Here we report the development of a loop-mediated isothermal amplification (LAMP) based method to detect SARS-CoV-2 genes ORF8 and N directly from pharyngeal swab samples. The established reverse transcription LAMP (RT-LAMP) assay detects SARS-CoV-2 directly from pharyngeal swab samples without previous time-consuming and laborious RNA extraction. The assay is sensitive and highly specific for SARS-CoV-2 detection, showing no cross reactivity when tested on 20 other respiratory pathogens. The assay is 12 times faster and 10 times cheaper than routine reverse transcription real-time polymerase chain reaction, depending on the assay used.

**Conclusion:**

The fast and easy to handle RT-LAMP assay amplifying specifically the genomic regions ORF8 and N of SARS-CoV-2 is ideally suited for POCT at e.g. railway stations, airports or hospitals. Given the current pandemic situation, rapid, cost efficient and onsite methods like the here presented RT-LAMP assay are urgently needed to contain the viral spread.

## Introduction

The emergence of the severe acute respiratory syndrome coronavirus 2 (SARS-CoV-2) has rapidly become a global public health emergency since its outbreak in Wuhan, China in December 2019 [1]. SARS-CoV-2 is an enveloped, non-segmented, single-stranded, positive-sense RNA virus similar to severe acute respiratory syndrome coronavirus (SARS-CoV) and Middle East respiratory syndrome coronavirus (MERS-CoV) [1]. Epidemical data show that the virus has strong human-to-human transmission ability, and is spread mainly by droplets produced by coughing and sneezing. Even talking or simple breathing can be enough for transmission [2]. The life-threatening respiratory infections caused by this virus designated as corona virus disease 2019 (COVID-19) are spreading worldwide [3]. Typical clinical symptoms of COVID-19 patients are fever, dry cough, breathing difficulties (dyspnoea), headache and pneumonia. COVID-19 may result in progressive respiratory failure owing to alveolar damage and even death [1]. As of October 1st 2020, more than 33,500,000 cases of infection have been confirmed, including over 1 million deaths [4]. To slow down and limit the spread, it is crucial to rapidly identify infected people, followed by strict public health measures. The current recommended testing method for potentially infected people by the Center of Disease Control and Prevention (CDC) and other relevant departments worldwide is the detection of SARS-CoV-2 nucleic acid via reverse transcription real-time polymerase chain reaction (RT-qPCR). The general methodology is based on reverse transcription of the viral RNA in a first step and subsequent PCR-amplification of the resulting cDNA in a second step to reach fluorescently detectable nucleic acid levels. In late spring 2020, RT-qPCR based methods were established for SARS-CoV-2 testing by multiple research and disease control centres around the globe [5]. Although RT-qPCR methods are the gold standard for the detection of nucleic acids of viral pathogens due to their high sensitivity and specificity, there are still some caveats. To perform this method, one needs a molecular biological laboratory facility with access to highly specialised laboratory instruments along with highly trained personnel. Especially the need for high purity nucleic acid extractions limits the testing capacity of this method, as this requires laborious and time-consuming RNA extraction and purification steps from patients’ swab samples, followed by long RT-qPCR run times. The pandemic situation is pushing even the developed nations to levels, where they are struggling to ensure rapid and effective testing for every suspected case. However, RT-qPCR assays are hardly able to satisfy the current demands of testing large numbers of persons (i. e. suspected patients and asymptomatic patients as well as close contacts). There is an urgent demand for a rapid, simple and sensitive point-of-care testing (POCT) assay, which could be used at airports, railway stations and hospitals, particularly regional hospitals and medical centres in rural areas, to facilitate faster detection of SARS-CoV-2, which can reduce or avoid further spread.

Loop-mediated isothermal amplification (LAMP) is a technology that provides nucleic acid amplification in a short time using 4 to 6 specially designed primers and a DNA polymerase with chain displacement activity [6]. The specialised version of DNA polymerase with strand displacement activity bypasses the need of DNA denaturation by heat. Since the LAMP method only needs one constant temperature (usually 65 °C), the performance device can be simpler and therefore cheaper and smaller than a thermal cycler. If the template is RNA, the amplification reaction can be accomplished in one step by adding a reverse transcriptase, and is therefore called reverse transcription LAMP (RT-LAMP) [6]. Compared to commonly used polymerases for PCR, the *Bacillus stearothermophilus* (*Bst*) polymerase used in LAMP is highly tolerable to inhibitors present in clinical samples [7, 8].

In this study, we report a novel reverse transcription loop-mediated isothermal amplification (RT-LAMP) detecting SARS-CoV-2 reliably in clinical swab samples without a laborious RNA extraction step.

## Materials and methods

### SARS-CoV-2 handling in cell culture

SARS-CoV-2 isolate named LGL-SCoV2-I1 was obtained from a patient with laboratory-confirmed diagnosis of SARS-CoV-2 infection as described recently [9]. Briefly, the patient’s pharyngeal swab sample was filtered through a 0.45 µm Minisart® syringe filter (Sartorius Stedim Biotech, Goettingen, Germany) and inoculated on a monolayer of Vero E6 cells (ATCC® CRL-1586™) for 5 days at 37 °C in a 5% carbon dioxide atmosphere until typical cytopathic effect (CPE) was visible. Vero E6 cells were grown in DMEM growth media supplemented with 10% heat inactivated fetal calf serum, 1% penicillin–streptomycin solution (10,000 U/mL, Gibco, Invitrogen, Carlsbad, USA) and 1% Fungizone (250 µg/mL, Gibco, Invitrogen, Carlsbad, USA). After verifying the integrity of the SARS-CoV-2 isolate in the cell culture supernatant by RT-qPCR utilizing the RealStar® SARS-CoV-2 RT-PCR Kit 1.0 (#821005, Altona Diagnostics, Hamburg, Germany), the isolate LGL-SCoV2-I1 was further propagated in Vero E6 cells. Viral stocks were generated from infected cell culture supernatants 36 h post infection and stored at − 80 °C until further usage.

### Conventional RT-qPCR reaction

Several commercially available RT-qPCR kits based on fluorescently labelled hydrolysis probes are routinely used to test for SARS-CoV-2 at the Bavarian Health and Food Safety Authority (LGL). In this study, the commercial test kits ampliCube Coronavirus SARS-CoV-2 (#50143 (50144), Mikrogen Diagnostik, Neuried, Germany) and FTD SARS-CoV-2 (#FTD-114-96, Fast Track Diagnostics (A Siemens Healthineers Company), Esch-sur-Alzette, Luxembourg) were used following previous RNA extraction utilising a Maxwell 16 extraction robot together with the Maxwell 16 Blood DNA Kit (#AS1400, Promega GmbH, Mannheim, Germany). Reactions were performed in accordance to each manufacturer’s instructions, respectively, and carried out in a CFX96 real-time thermal cycler (Bio-Rad, Hercules, USA) or a Quantstudio 7 real-time thermal cycler (Thermo Fisher Scientific, Waltham, USA). CFX Maestro software (Bio-Rad, Hercules, USA; version 1.1) or QuantStudio™ Real-Time PCR software (Thermo Fisher Scientific, Waltham, USA) were used for data acquisition and analysis.

### RT-LAMP for SARS-CoV-2

Viral genes ORF8 and N were chosen as target regions for the RT-LAMP. For the detection of the N gene, which is targeted by many commercially available SARS-CoV-2 tests, primers designed by Zhang et al. were applied [10], whereas customised primers were designed using LAMP-Designer software (Premier Biosoft, Palo Alto, USA; V1.16) for the detection of the viral ORF8 gene (Table 1, Additional file 1: Figure S1). Because of the great sequence diversity of the gene ORF8 in SARS-CoV and SARS-CoV-2 genomes, ORF8 was considered a suitable target for specific LAMP primer design. When analysing patients’ swab samples, human RNase P POP7 was additionally targeted as an internal control (IC) to monitor the presence and quality of cellular material attached to the swab (Table 1). This IC was chosen to verify the correct collection of the clinical pharyngeal swab and to avoid potential possible false negative SARS-CoV-2 results. The RT-LAMP assay was carried out in a total volume of 25 µL consisting of 12.5 µL 2 × WarmStart LAMP Master Mix, 0.5 µL LAMP Fluorescent Dye (WarmStart® LAMP Kit (DNA & RNA), #E1700L, NEB, Ipswich, USA), 2.5 µL 10 × primer mix, 4.5 µL PCR grade water and 5 µL sample (consisting of either isolated RNA or heated swab sample, respectively). As non-template control (NTC) PCR grade water substituted the sample. Concentration of each primer in the 10 × primer mix were the following: 2 µM of each outer primer (F3 and B3), 16 µM of each inner primer (FIP and BIP) and 8 µM of each loop primer (LF and LB). Reactions were carried out in a Quantstudio 7 real time thermal cycler (Thermo Fisher Scientific, Waltham, USA) for the benefit of measuring in 96-well format during assay optimisation phase as well as on the Genie II instrument (OptiGene, Horsham, United Kingdom) as a suitable point-of-care testing device. Reaction conditions were 25 min at constant 63 °C, 65 °C or 67 °C with continuous fluorescence detection. Reactions were considered positive if sample amplification (fluorescence signal via intercalation of LAMP Fluorescent Dye into amplified product above a given threshold) was detected.

**Table 1:** Sequences of LAMP primers for the detection of SARS-CoV-2

Target gene	Primer	Sequence 5′–3′	Source
N	GeneN-A-F3	TGGCTACTACCGAAGAGCT	Zhang et al. [10]
	GeneN-A-B3	TGCAGCATTGTTAGCAGGAT	
	GeneN-A-FIP	TCTGGCCCAGTTCCTAGGTAGTCCAGACGAATTCGTGGTGG	
	GeneN-A-BIP	AGACGGCATCATATGGGTTGCACGGGTGCCAATGTGATCT	
	GeneN-A-LF	GGACTGAGATCTTTCATTTTACCGT	
	GeneN-A-LB	ACTGAGGGAGCCTTGAATACA	
ORF8	ORF8-F3	ACTTGTCACGCCTAAACG	This study
	ORF8-B3	CTACCCAATTTAGGTTCCTGG	
	OFR8-FIP	AGGACACGGGTCATCAACTACAAGCTGCATTTCACCAAGAA	
	ORF8-BIP	AGGAGCTAGAAAATCAGCACCTATGGGTGATTTAGAACCAGC	
	ORF8-LF	TGGTTGATGTTGAGTACATGAC	
	ORF8-LB	AATTGAATTGTGCGTGGATGAG	
RNase P POP7	RNaseP-POP7-F3	TTGATGAGCTGGAGCCA	Curtis, Morrison et al. [14]
	RNaseP-POP7-B3	CACCCTCAATGCAGAGTC	
	RNaseP-POP7-FIP	GTGTGACCCTGAAGACTCGGTTTTAGCCACTGACTCGGATC	
	RNaseP-POP7-BIP	CCTCCGTGATATGGCTCTTCGTTTTTTTCTTACATGGCTCTGGTC	
	RNaseP-POP7-LF	ATGTGGATGGCTGAGTTGTT	
	RNaseP-POP7-LB	CATGCTGAGTACTGGACCTC	

The table shows the sequences of the LAMP (loop-mediated isothermal amplification) primers used in this study. The primers for amplification of SARS-CoV-2 gene N and human RNase P POP7 were previously published. Primers for SARS-CoV-2 amplification were specifically designed for this study

### SARS-CoV-2 quantification using reverse transcriptase droplet digital PCR

The initial genome copy number of SARS-CoV-2 was determined using reverse transcriptase droplet digital PCR (RT-ddPCR). For this purpose, RNA was isolated from a SARS-CoV-2 infected Vero cell culture using the QIAamp Viral RNA Mini Kit (#1020953, Qiagen, Hilden, Germany) according to the manufacturer´s instructions. RT-ddPCR was performed using primers and probe published by the Chines CDC [11, 12] targeting the N gene in combination with the One-Step RT-ddPCR Advanced Kit for Probes (#1864022, Bio-Rad, Hercules, USA). A total of 2 μL of virus RNA was added to 18 μL of ddPCR reaction mix containing 1 × ddPCR supermix (#1864022, Bio-Rad, Hercules, USA) and primers and probes dissolved in PCR grade water. PCR grade water served as non-template control. Droplets were generated using 8-well cartridges in a droplet generator (Bio-Rad, Hercules, USA) and then transferred to a 96-well plate using a multichannel pipette. End-point PCR was performed using a T100 thermal cycler (Bio-Rad, Hercules, USA) under the following cycling conditions: 60 min reverse transcription at 45 °C, 10 min initial denaturation at 95 °C, 60 cycles of 95 °C for 30 s and 59 °C for 2 min, and finally 10 min at 98 °C. A heating ramp rate of 1 °C per second was applied during all cycling steps. After amplification, droplet separation, counting and fluorescence measurement were performed in the QX100 Droplet Reader (Bio-Rad, Hercules, USA). The QuantaSoft software (Bio-Rad, Hercules, USA; version 1.7.4) was used for data acquisition and analysis.

### Limit of detection

The limit of detection (LOD) of the RT-LAMP assay was determined according to guidelines for the single-laboratory validation of qualitative real-time PCR methods of the Federal Office Of Consumer Protection and Food Safety (Bundesamt für Verbraucherschutz und Lebensmittelsicherheit (BVL)). Quantified virus RNA was diluted in PCR grade water containing 20 ng/µL herring sperm background DNA (Promega GmbH, Mannheim, Germany) to generate target concentrations of 1000 copies/µL, 200 copies/µL, 100 copies/µL, 20 copies/µL, 10 copies/µL, 4 copies/µL, 2 copies/µL, 1 copy/µL and 0.2 copies/µL, respectively. Dilutions of 1000 copies/µL and 200 copies/µL were analysed in six replicates, whereas 12 replicates were applied for dilutions between 100 copies/µL and 0.2 copies/µL. The LOD was defined as the last sample target concentration at which all 12 replicates were tested positive for the respective target.

### Specificity of the assay

To determine the specificity of the SARS-CoV-2 RT-LAMP assay, SARS-CoV Frankfurt 1 RNA [#004N-02005, European Virus Archive (EVAg)] [13] and nucleic acid extracts from 20 different samples from respiratory pathogens (Respiratory Verification Panel, #NATRVP-QIA, ZeptoMetrix Corporation, Buffalo, USA) were tested. Nucleic acid was extracted from Respiratory Verification Panel samples utilising a Maxwell 16 extraction robot together with the Maxwell 16 Blood DNA Kit (#AS1400, Promega GmbH, Mannheim, Germany).

### Pre-treatment of patients’ swab samples for RT-LAMP

Positive and negative SARS-CoV-2 routine samples (Positive samples 1–5 and Negative samples 1–5), tested at the Bavarian Health and Food Safety Authority, were used to verify the optimal pre-treatment temperature to inactivate virus particles and make SARS-CoV-2 RNA accessible for amplification. Positive samples 1–5 and Negative samples 3, 4 and 5 were of the Traswab type (Additional file 2: Table S1, Swab 1), while Negative sample 1 was of the Virocult type (Additional file 2: Table S1, Swab 3) and Negative sample 2 of the eSWAB type (Additional file 2: Table S1, Swab 2). The same samples were also used for colorimetric LAMP and establishment of the RT-LAMP assay at the Genie II instrument. Positive SARS-CoV-2 test results were verified with ampliCube Coronavirus SARS-CoV-2 test (#50143 (50144), Mikrogen Diagnostik, Neuried, Germany), which was validated in a recent validation study of different commercially available molecular assays for detection of SARS-CoV-2 RNA at the Bavarian Health and Food Safety Authority (manuscript in preparation). Original swab samples were vortexed thoroughly, transferred into low bind 1.5 mL reaction tubes and heated at 80 °C, 85 °C, 90 °C or 95 °C for 5 min in a pre-heated heating block. Heated swabs were vortexed and 5 µL of each sample were pipetted directly into the RT-LAMP reaction. All potentially infectious work was performed in a safety cabinet in accordance to the respective bio safety standards.

### Colorimetric RT-LAMP for SARS-CoV-2

In addition to the detection via fluorescence, the WarmStart Colorimetric LAMP 2 × Master Mix (#M1800S, NEB, Ipswich, USA) was used under the same conditions as described above with 5 µL of the same heated swab samples for simpler read-out based on colour change by eye only. On the contrary to fluorescent signal detection, the method is based on the change of colour from pink to yellow following the change of pH by enormous DNA amplification in positive samples. The reactions took place in a total volume of 25 µL, consisting of 12.5 µL WarmStart Colorimetric LAMP 2 × Master Mix (#M1800S, NEB, Ipswich, USA), 2.5 µL 10 × primer mix, 5 µL PCR grade water and 5 µL heated swab sample. As non-template control (NTC) PCR grade water substituted the sample. Concentration of each primer in the 10 × primer mix were the following: 2 µM of each outer primer (F3 and B3), 16 µM of each inner primer (FIP and BIP) and 8 µM of each loop primer (LF and LB). Reactions were carried out in a Quantstudio 7 real time thermal cycler (Thermo Fisher Scientific, Waltham, USA). Reaction conditions were 25 min at a constant temperature of 67 °C. Reactions were considered positive if yellow colour was visible.

### Suitability evaluation of different medical swabs and viral transport media for RT-LAMP

To test the potential influence of different medical swabs and their viral transport media, seven of the most frequently used swabs in routine testing at the Bavarian Health and Food Safety Authority since March 2020 were chosen and systematically analysed (Additional file 2: Table S1). In order to obtain comparable negative samples, pharyngeal samples were taken from the same SARS-CoV-2 negative person with each tested swab and viral transport media combination. The only tested dry swab, Swab 7 (#155C, Copan), was incubated in 1.6 mL of 0.9% NaCl for 30 min directly after taking the pharyngeal sample prior to thorough vortexing. 10 µL of a 1/100 dilution of SARS-CoV-2 infected Vero cell culture supernatant in PCR grade water was heat inactivated and subsequently added to each of the seven sample tubes to generate comparable positive samples. The seven different swab samples were tested for positive or negative LAMP reaction monitoring the reaction time until positive results.

## Results

### Optimising the RT-LAMP assay

**Fig. 1:**
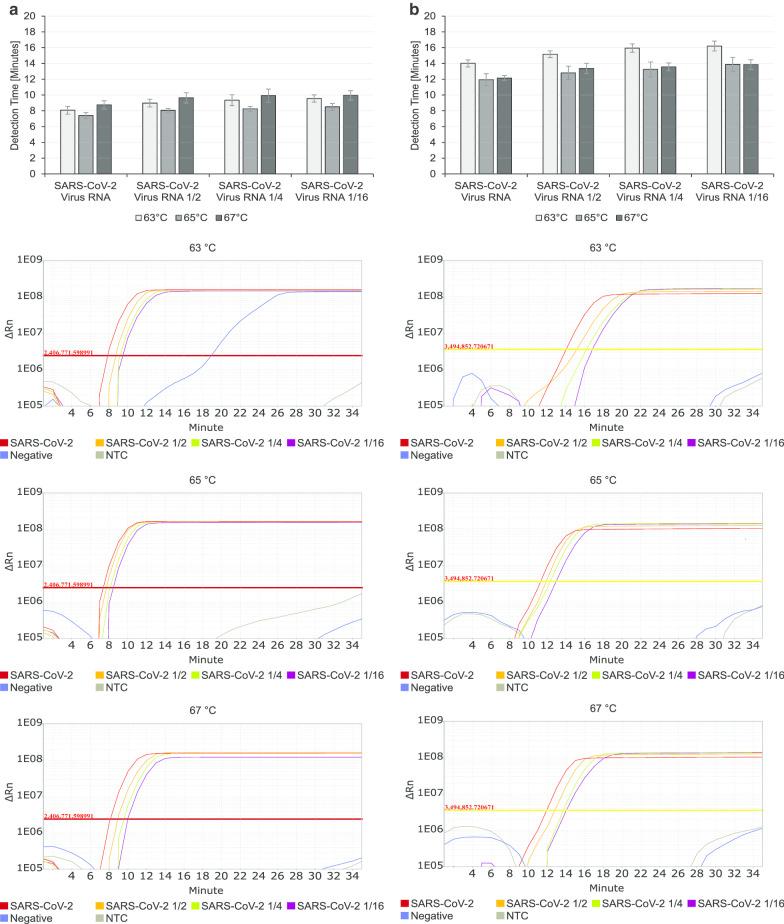
Optimising RT-LAMP temperature. RT-LAMP reactions for ORF 8 (**a**) and N (**b**) detection of SARS-CoV-2 were incubated at 63 °C, 65 °C and 67 °C for 35 min. Extracted RNA from SARS-CoV-2 infected Vero cell culture supernatant was tested along with 1/2, 1/4 and 1/16 dilutions in PCR grade water. As negative controls, pooled negatively tested patient RNA (Negative) and water (NTC) were used. All experiments were performed in triplicates three times over. Amplification Plots show representative results of nine measurements per sample

To screen for the optimum temperature of the designated RT-LAMP, the reaction was incubated at three different temperatures (63 °C, 65 °C, 67 °C) for 35 min. For this screening, initially 1/100 diluted isolated RNA from SARS-CoV-2 infected Vero cell culture supernatant in PCR grade water together with additional dilutions (1/2, 1/4, 1/16) were used as positive samples. As negative controls pooled RNA from routinely tested negative patient swabs and water were used. From the three tested temperatures, 67 °C generated the most reliable and accurate results (Fig. 1). Especially for the detection of gene N, 67 °C yielded very fast positive signals (Fig. 1b) whereas there were no strong differences in the detection time for ORF8 (Fig. 1a). Decisive however, to choose 67 °C as the optimum temperature for the RT-LAMP assay was the increasing stability and specificity of the assay with higher temperatures. Whereas at 63 °C and 65 °C few of the negative controls showed late positive signals for ORF8 detection (Fig. 1a), no false positive results occurred at 67 °C, while the amplification curves ran more stable and accurate (Fig. 1).

### Sensitivity of the RT-LAMP assay

Isolated RNA from SARS-CoV-2 infected Vero cell culture supernatant was quantified using RT-ddPCR and serially diluted to determine the LOD. Each dilution was analysed in six or 12 replicates. When using virus RNA concentrations of 1000–100 copies/µL all replicates were positive in both genes ORF8 and N. At a concentration of 20 copies/µL, 9 out of 12 replicates were positive in both targets (Additional file 3: Figure S2). Hence, the LOD for both SARS-CoV-2 detection targets was determined as 100 copies/µL, which corresponds to 500 copies per RT-LAMP reaction.

### Specificity of the RT-LAMP assay

The specificity of the RT-LAMP assay was evaluated using 21 human respiratory pathogens. Specificity testing revealed no unspecific signals for the targeted genes ORF8 and N in the SARS-CoV-2 genome (Table 2, Additional file 4: Figure S3). Strikingly, none of the tested respiratory validation panel materials resulted in false positive amplification signals, not even SARS-CoV, underlining the high specificity of the developed assay. Positive amplification signals originated solely from the respective RNA isolated from SARS-CoV-2 infected Vero cell culture supernatant (Table 2, Additional file 4: Figure S3).

**Table 2:** Specificity test of RT-LAMP detecting genes ORF8 and N of SARS-CoV-2

Organism	ORF8	N
Adenovirus type 3	−	−
*Bordetella pertussis*	−	−
*Chlamydia pneumoniae*	−	−
Coronavirus 229E	−	−
Coronavirus HKU-1	−	−
Coronavirus NL63	−	−
Coronavirus OC43	−	−
Influenza A 2009 H1N1pdm	−	−
Influenza A H1N1	−	−
Influenza A H3N2	−	−
Influenza B	−	−
*Mycoplasma pneumoniae*	−	−
Metapneumovirus 8	−	−
Parainfluenza virus type 1	−	−
Parainfluenza virus type 2	−	−
Parainfluenza virus type 3	−	−
Parainfluenza virus type 4	−	−
Rhinovirus 1A	−	−
RSV A	−	−
SARS-CoV	−	−
SARS-CoV-2	+	+

Nucleic acid extracts of 21 different respiratory pathogens were analysed with RT-LAMP detecting ORF8 and gene N from SARS-CoV-2. Successful amplification is indicated with “+”, whereas “−” indicates a negative result

### RT-LAMP assay detecting SARS-CoV-2 directly from patients’ swabs

**Fig. 2:**
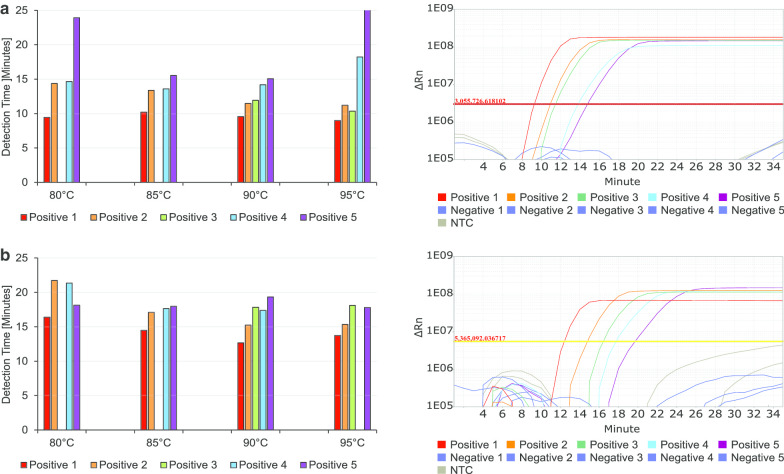
Optimising pre-treatment of patient’s swab samples for direct usage in RT-LAMP. Positive sample 1–5 and Negative sample 1–5 were pre-heated at 80 °C, 85 °C, 90 °C and 95 °C for 5 min before being directly pipetted into RT-LAMP for SARS-CoV-2 targeting OFR8 (**a**) and N (**b**). All experiments were performed three times in duplicates. Amplification Plots show representative results for six measurements per sample

For faster and easier detection of SARS-CoV-2 in patients’ swab samples, it would be beneficial to avoid the highly specialised and time-consuming RNA extraction and purification step. Unfortunately, high purity of RNA is crucial to all common detection systems like RT-qPCR. However, we found this step unnecessary and dispensable to perform accurate and sensitive RT-LAMP for detecting SARS-CoV-2. We tested a pre-treatment for pharyngeal swab samples from SARS-CoV-2 suspected patients. A simple pre-heating step at 90 °C for 5 min prior to testing in RT-LAMP worked sufficiently thereby bypassing intermittent shortages of RNA extraction chemicals. To optimise detection results to the fastest and most accurate outcome, we tested heating swab samples at four different temperatures from 80 to 95 °C for 5 min each in a pre-heated heating block. The heated samples were directly pipetted into the prepared RT-LAMP reaction and run for 25 min at 67 °C detecting fluorescent signal from integrating LAMP Fluorescent Dye (Fig. 2). Positive swab samples 1–5, which were tested positive for SARS-CoV-2 in routine RT-qPCR, and Negative swab samples 1–5, which were negatively tested, were used. Pre-heating the swabs at 90 °C for 5 min before directly pipetting them into the RT-LAMP reaction resulted in the best combination of low detection time and specific amplification of SARS-CoV-2 positive samples (Fig. 2). As there is no control in the routinely used RT-qPCR for human material in the patient’s swab at all, an additional target was analysed to proof the validity of the swab itself within the designed RT-LAMP assay. For this purpose, primer for human RNase P POP7 were utilised as an internal control (IC) in patient swab samples (Table 3) [14]. The test showed that RNase P was positive in all ten tested swab samples, proofing the validity of the tested swabs. Positive signals for SARS-CoV-2 were generated accurately and in less than 20 min running time of the assay, even for Positive sample 5 with a relatively late Cq value in RT-qPCR (Cq 30, Table 3). We additionally tested to read-out results using a colorimetric approach. Therefore, WarmStart Colorimetric LAMP 2 × Master Mix was used according to the manufacturer’s instructions with 5 µL of heated swab samples Positive 1–5 and Negative 1–5. The read-out was neither as sensitive as the fluorescent one, nor as accurate (Additional file 5: Figure S4). The observable colour change is based on change in pH during DNA amplification. Therefore, it seems likely that false positive signals in the test (Additional file 5: Figure S4, Negative 1 and Negative 2) could be due to a high sensitivity of the pH dependent read-out to different viral transport media or pharyngeal compounds from the patients themselves. There are various compounds imaginable that could influence pH in pharyngeal swab samples from bacterial superinfection of the patient to coffee consumption directly prior to testing. Colorimetric read-out is therefore not well suited and not recommended for the here described workflow with direct detection of SARS-CoV-2 in RT-LAMP. However, the colorimetric read-out is shown to be well suited when working with isolated RNA instead of direct swab samples [15–17].

**Table 3:** Quantification Cycle (Cq) values of RT-qPCR compared to detection times of RT-LAMP detecting SARS-CoV-2 in pharyngeal swab samples

Pharyngeal swab	Cq value RT-qPCR Orf1a	Detection time RT-LAMP ORF8	Detection time RT-LAMP N	Detection time RT-LAMP RNase P (IC)
Positive 1	18.40	9.55	12.67	22.78
Positive 2	24.13	11.48	15.24	23.22
Positive 3	28.97	11.93	17.84	19.34
Positive 4	29.26	14.19	17.39	20.30
Positive 5	30.47	15.05	19.34	20.98
Negative 1				20.97
Negative 2				21.06
Negative 3				21.69
Negative 4				23.96
Negative 5				15.18

Ten pharyngeal swab samples were routinely tested for COVID-19 using ampliCube Coronavirus SARS-CoV-2 kit acquired from Mikrogen Diagnostik, where Orf1a serves as specific target for SARS-CoV-2. Positive samples 1–5 and Negative samples 1–5 were pre-heated at 90 °C for 5 min and directly tested for SARS-CoV-2 in RT-LAMP. The targets ORF8 and N detect SARS-CoV-2, while RNase P is used as internal control (IC) for proof of human material in the swab

### Suitability of different pharyngeal swabs and viral transport media for RT-LAMP assay

**Fig. 3:**
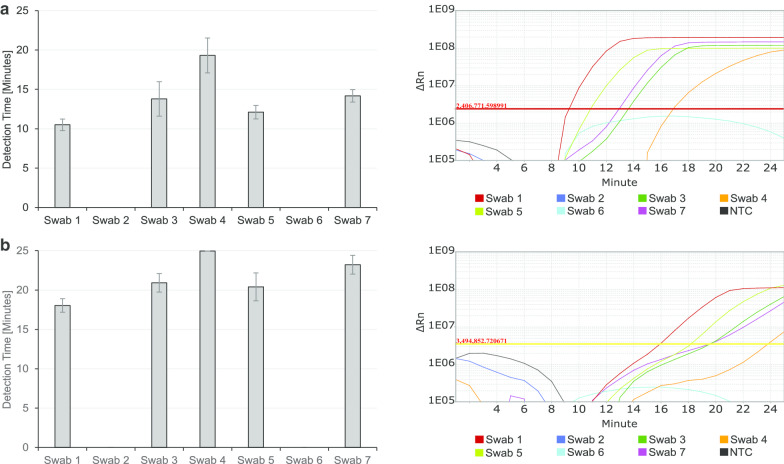
Suitability of different swab types available for direct SARS-CoV-2 detection in RT-LAMP. Seven of the most commonly used swab types were used to test one SARS-CoV-2 negative person. 10 µL of heat-inactivated virus was added to each swab type. Samples were heated to 90 °C for 5 min before their addition to RT-LAMP reaction detecting ORF8 (**a**) and N (**b**). All experiments were performed three times in triplicates. The Amplification plots show representative amplification curves for nine measurements per sample

Since we used the sample material directly and did not extract RNA first, possible influences of the swab material and transport media are of great importance for the outcome of our RT-LAMP assay. To test for the suitability of different pharyngeal swabs and their respective viral transport media, one SARS-CoV-2 negative person was tested with seven different swab types, including pharyngeal material in the test. To compare positive results in the different viral transport media, 10 µL of a heat-inactivated viral culture supernatant were added to each test vial irrespective of the different liquid volumes in the seven different swabs. Initial viral transport media volumes in the different swabs varied between roughly 1.5 mL up to 5 mL. With these preparations, ideally comparable initial situations were created to accurately compare the different swab materials and viral transport media for their impact on direct SARS-CoV-2 testing in RT-LAMP. Swabs were simultaneously heated at 90 °C for 5 min and 5 µL of each swab sample were directly pipetted into RT-LAMP reactions for ORF8, N and RNase P. After incubation for 25 min at 67 °C major differences between the tested samples not only in detection time, but even in the overall potential to detect SARS-CoV-2 at all with this workflow were detected. Two of the seven tested swab materials, Swab 2 and Swab 6 (Additional file 2: Table S1, eSWAB and Virus Sample Stabilizer), showed no amplification curve and it was not possible to detect SARS-CoV-2 in these samples (Fig. 3). The Virus Sample Stabilizer swab type (Additional file 2: Table S1, Swab 6) had an extremely high content of salt, which was already visible by a white salt crust around the lid. However, there are obviously some inhibiting factors in these two negatively tested swabs or their used viral transport media prevents successful RT-LAMP detection of the virus. It is highly recommended not to use these two types of swabs for the direct SARS-CoV-2 testing in the here described RT-LAMP assay. The other five swab types are basically well suited for this assay, as all five swab samples tested positive for SARS-CoV-2, even though detection time differed up to 7–8 min from earliest to latest positive test results (Fig. 3). When comparing those five swabs, Swab 1 (Additional file 2: Table S1, Transwab) is highly recommended to use for this direct SARS-CoV-2 RT-LAMP due to a low detection time and a high amplification signal.

**Fig. 4:**
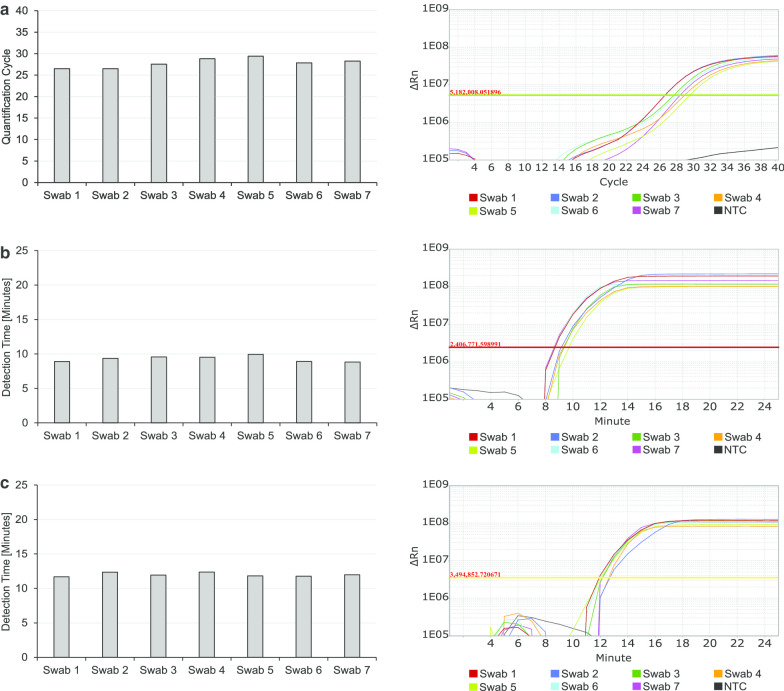
Effects of different swab types on RT-qPCR and RT-LAMP results with isolated RNA. Seven of the most commonly used swab types were used to test one SARS-CoV-2 negative person. 10 µL of heat-inactivated virus was added to each swab type. RNA was extracted and purified before being analysed for SARS-CoV-2 in RT-qPCR (Siemens Kit) targeting Orf1a (**a**) and RT-LAMP targeting ORF8 (**b**) and N (**c**)

**Fig. 5:**
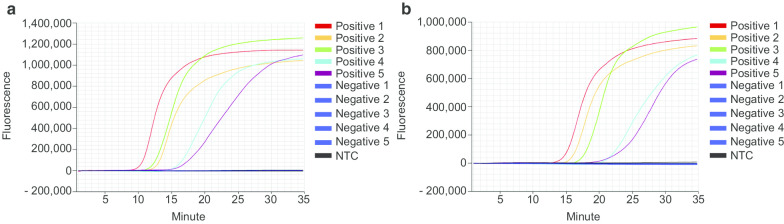
Direct detection of SARS-CoV-2 for POCT with the Genie II instrument. Positive samples 1–5 and Negative samples 1–5 were pre-heated at 90 °C for 5 min before being directly pipetted into RT-LAMP for SARS-CoV-2 targeting ORF8 (**a**) and N (**b**). All experiments were performed three times. Amplification Plots show representative results for three measurements per sample

Additionally, RNA was extracted and purified to test, if the different swab types also influence RT-qPCR results and RT-LAMP results for SARS-CoV-2 detection after several washing steps during RNA isolation. Extracted RNA was tested in RT-qPCR and RT-LAMP as previously described. It is not surprising, that after removal of the different viral transport media during the procedure of RNA extraction, the differences in detection time are less striking when analysing extracted and purified RNA. In RT-qPCR and also in RT-LAMP using extracted RNA from the seven tested swabs and viral transport media, all tests showed positive signals for SARS-CoV-2 (Fig. 4). Nevertheless, some inhibiting factors or volume effects due to the different viral transport media volumes in the different original swab vials still remain (Fig. 4). For RT-qPCR the difference between Swab 1 (Additional file 2: Table S1, Transwab), with the lowest Cq value (26.49), and Swab 5 (Additional file 2: Table S1, Liquid Amies Midia—virus transport kit), with the highest Cq value (29.41), is about 3 Cq values (Fig. 5a). As exactly the same amount of virus was added to all different swab types, this is most likely the effect of different volumes of viral transport media diluting the virus in the original swab vial or inhibiting factors remaining after RNA extraction. For RT-LAMP analysis the difference between the lowest detection time (8.82 min for ORF8, 11.69 min for N) and the highest detection time (9.94 min for ORF8 and 12.39 min for N) in both targets for SARS-CoV-2 is about 1 min and therefore negligible (Fig. 4b, c).

### Comparison of RT-LAMP assay to RT-qPCR Cq values

After the determination of the optimal conditions for SARS-CoV-2 detection with RT-LAMP, 40 positive tested swab samples from the routine SARS-CoV-2 testing at the Bavarian Health and Food Safety Authority were tested in RT-LAMP. All samples were of the Transwab type (Additional file 2: Table S1, Swab 1) and tested without prior RNA extraction directly after heating swab samples at 90 °C for 5 min. 100% of tested swab samples with a RT-qPCR Cq value below 25 were tested positively in RT-LAMP (Table 4). As single swab samples with RT-qPCR Cq values between 25 and 30 showed false negative results, the overall sensitivity of RT-LAMP for RT-qPCR Cq values below 30 is 93% for the target gene ORF8 and 96% for gene N. Targeting the gene ORF8 of SARS-CoV-2, the sensitivity of RT-LAMP for RT-qPCR Cq values until 35 still is 91%. Overall, RT-LAMP detecting SARS-CoV-2 nucleic acid directly from swab samples without prior RNA extraction is 100% accurate for RT-qPCR Cq values below 25. With more than 90% correctly detecting positive swab samples with RT-qPCR values until 30 for gene N and even until Cq 35 for ORF8, sensitivity of the here described RT-LAMP assay directly analysing swab samples should be sufficient for POCT purposes.

**Table 4:** Comparison of RT-qPCR Quantification Cycle (Cq) values to RT-LAMP positive results

Cq value RT-qPCR	Positive result RT-LAMP ORF8	Positive result RT-LAMP N
< 20	5 out of 5 (100%)	5 out of 5 (100%)
20–25	9 out of 9 (100%)	9 out of 9 (100%)
25–30	12 out of 14 (86%)	13 out of 14 (93%)
30–35	6 out of 7 (86%)	0 out of 7 (0%)
> 35	2 out of 5 (40%)	0 out of 5 (0%)

40 pharyngeal swab samples were routinely tested for COVID-19 using ampliCube Coronavirus SARS-CoV-2 kit acquired from Mikrogen Diagnostik, where Orf1a serves as specific target for SARS-CoV-2. The same pharyngeal swab samples were pre-heated at 90 °C for 5 min and directly tested for SARS-CoV-2 in RT-LAMP. The targets ORF8 and N detect SARS-CoV-2 nucleic acid in the swab samples

### Transferability of SARS-CoV-2 RT-LAMP to point-of-care testing (POCT) instruments

All method optimising experiments were carried out with a Quantstudio 7 real-time thermal cycler for the benefit of 96-well plate measurements. However, such highly specialised equipment is not well suited for urgently needed POCT. Therefore, the RT-LAMP assay for detecting SARS-CoV-2 nucleic acid directly in patients’ swab samples was successfully established at the Genie II instrument (OptiGene, Horsham, United Kingdom) (Fig. 5), a device especially designed for POCT requirements. The Genie II is a compact, lightweight and robust instrument suitable for use in the field or laboratory. It was specifically designed to run any isothermal amplification method that employs target detection by fluorescence measurement. The instrument boasts low power requirements and includes a rechargeable Lithium-Polymer battery that can keep it running for a full working day. The Genie II combines isothermal amplification and fluorescence detection with a small and lightweight appearance not necessarily dependent on power supply at the point-of-care. With this instrument and the fast and easy workflow of this RT-LAMP assay, SARS-CoV-2 testing could be carried out directly where it is needed the most, at railway stations, airports, hospitals or at the general practitioner’s office next door.

## Discussion

**Fig. 6:**
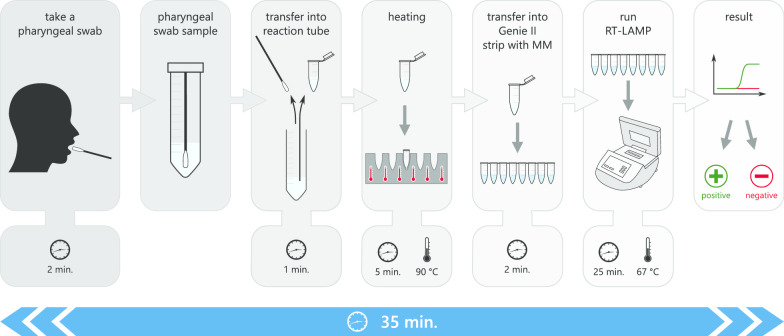
Workflow of the presented RT-LAMP assay testing direct patients’ swab samples for SARS-CoV-2 (*MM* master mix)

RT-LAMP without prior RNA extraction is a very easy and rapid possibility to test for SARS-CoV-2 infections. It is applicable at any point-of-care to test directly on site without any costly and highly specialised equipment or personnel necessary. Nevertheless, RT-LAMP assays reaching higher sensitivity in detecting SARS-CoV-2 have previously been published. These studies reach very high sensitivities starting from 100 copies [18, 19] of viral RNA per reaction down to 10–20 copies [17, 20], detecting even as low as two or three copies [15, 16] of viral RNA per reaction. Different methods in science are in general used for the determination of gene or genome copy numbers. This fact could lead to the diverse results in sensitivity measurements for the different cited RT-LAMP methods. While RT-ddPCR, which was used to determine copy numbers for LOD assessment in the present study, gives the exact number of amplifiable copy numbers of the specific target, determination of copy numbers by molecular weight is more an estimation of the actual number and can lead to slightly inaccurate assumptions. Even slight differences in the determination of the copy number in a sample could have great impact onto the final LOD assessment due to the utilised serial dilutions derived from the sample. These dilutions are based on the initially defined copy number and amplify a possible small initial variation to huge differences in sensitivity measurement. However, the more important point to sensitivity in this study is the comparison of actual Cq values of routinely tested swab samples measured via RT-PCR to the detectability of these samples in the here described RT-LAMP. The comparison to RT-qPCR tests currently used to diagnose COVID-19 all over the world is crucial for the decision if a new testing method can be categorized as sufficiently sensitive to serve the pandemic situation’s need for cost-efficient and fast testing of enormous numbers of people. The very recent publication by Dao Thi et al. [21] determines sensitivity of their RT-LAMP method in comparison to RT-qPCR Cq values instead of using copy numbers in terms of a LOD assessment. Working with extracted purified RNA, they reach a sensitivity of 97.5% for samples with RT-qPCR Cq values until 30. The same range of sensitivity is given by another study with 97.62% positive percent agreement in RT-LAMP detecting SARS-CoV-2 [22]. However Thi et al. [21] tested a similar pre-treatment condition for using direct pharyngeal swab samples and avoid RNA extraction in RT-LAMP, but utilised the less suitable colorimetric read-out, with lower sensitivity yield. Without prior RNA extraction using direct swab samples after a preheating step, the study yields 86% sensitivity for samples with a Cq value below 30 [21]. Given this data, the sensitivity of the here presented RT-LAMP assay for the detection of SARS-CoV-2 is within the current range of sensitivity yield for POCT purposes. Using the Transwab swab type for direct analysis of patients’ pharyngeal swab samples without prior RNA extraction is crucial to the success of the here described RT-LAMP assay. High salt concentrations in different viral transport media for example, could interfere with nucleic acid folding and loop formation, inhibiting RT-LAMP reaction [23]. Just as specific components of every viral transport media, the different volumes of viral transport media in the different available swab types influence the feasibility and efficiency of the RT-LAMP assay. Swab types with higher starting volumes of viral transport media will dilute the virus content obtained with the swab in comparison to lower starting volumes of viral transport media. The here shown differences between different swab types testing the exact same sample conditions highlight the importance of careful consideration of different swab types for specific purposes. During the pandemic situation, shortcomings in the availability of these swabs could limit the feasibility of this method. However, this study showed dry swabs as possible alternatives, even though they are slightly less suitable for direct detection of SARS-CoV-2. It was shown that detection from dry swab samples resulted in longer detection times, which might lead to lower sensitivity. Nevertheless, the broad availability of dry swabs along with their convenient shipping and storage conditions may outweigh the negative aspects in times of need. Despite the common practice in describing time requirements of new methods with only naming the pure run time of analyses, we describe exactly the time needed from taking the test sample of the prospective patient until the final result. Many time specifications of tests ignore transportation times or sample preparation until the actual measurement can be performed. The time span required for the whole RT-LAMP assay is only 35 min, every step considered (Fig. 6). Transportation times to highly specialised and equipped laboratories as well as for RNA extraction procedures are not incurring. This is more than 12 times faster than routinely performed RT-qPCR, as this method takes 6–7 h from the time of arrival at the laboratory institution until results can be reported [24]. Transportation times due to huge differences are already neglected in this study. The here described RT-LAMP assay is even faster than one of the fastest diagnostic methods for detecting SARS-CoV-2 nucleic acid on the market. The GeneXpert System from Cepheid returns test results in about 50 min while being more than 25 times more expensive than RT-LAMP, which costs less than 2 € per single reaction. Of note, the costs of routinely used RT-qPCR are also about 10 times higher than a single reaction in RT-LAMP, depending on the RT-qPCR kit used. In addition to the low costs per reaction, the one-time price for purchasing the here described all in one stand-alone point-of-care device is about 10,000 €, which is more than reasonable for setting up test centres at airports or railway stations in developed countries. This is still about seven times cheaper than a routinely used real-time thermal cycler needed for RT-qPCR. To reduce the costs for equipment even further, the here developed method could also be carried out in a simple water bath or heating block together with the read-out as an end-point fluorescent measurement in a plain fluorimeter. To monitor the fluorescence signal development, the samples could be analysed at 5 min, 15 min and 25 min time points during RT-LAMP with minimum extra time needed. End-point fluorimeters are already available starting with 1000 €.

## Conclusion

In conclusion, the here described RT-LAMP assay is a very sensitive, specific, cheap, easy to perform and rapid method detecting SARS-CoV-2 nucleic acid directly in pharyngeal swab samples without prior RNA extraction. It is an ideal point-of-care testing possibility serving the enormous needs for fast and reliable diagnosis on site urgently required in this pandemic situation.

## Supplementary information

**Additional file 1: Figure S1.** Positions of LAMP primers in SARS-CoV-2 genome. LAMP primers of genes ORF8 (A) and N (B) aligned to the SARS-CoV-2 reference sequence. Arrowheads indicate orientation of the primer. Primers F1c and F2 together build the FIP primer, whereas primers B1c and B2 together build the BIP primer of the different LAMP primer sets.

**Additional file 2: Table S1.** Seven different swab types for suitability test of different swab types for RT-LAMP.

**Additional file 3: Figure S2.** Determination of the sensitivity of the assay. Isolated RNA from SARS-CoV-2 infected Vero cell culture supernatant was quantified using RT-ddPCR and serially diluted to determine the LOD. 1000 copies/µL and 200 copies/µL dilutions were analysed in six replicates, while every lower dilution (1000–0.2 copies/µL) were analysed in 12 replicates. For both target genes ORF8 (A) and N (B) the LOD is 100 copies/µL as the last dilution where all 12 replicates are positive. For non-template control (NTC) PCR grade water substituted SARS-CoV-2 RNA.

**Additional file 4: Figure S3.** Specificity of the assay. SARS-CoV Frankfurt 1 RNA and nucleic acid extracts from 20 different samples from respiratory pathogens were tested with RT-LAMP targeting ORF8 (A) and N (B) to determine cross reactivity. For non-template control (NTC) PCR grade water was used.

**Additional file 5: Figure S4.** Colorimetric read-out of SARS-CoV-2 RT-LAMP. Positive samples 1–5 and Negative samples 1–5 were pre-heated at 90 °C for 5 min before being directly pipetted into RT-LAMP for SARS-CoV-2 targeting ORF8 and N. As internal control (IC) RNase P was additionally targeted. WarmStart Colorimetric LAMP 2x Master Mix (#M1800S, NEB, Ipswich, USA) was used by the manufacturer’s instructions with 5 µL of heated swab sample. For non-template control (NTC) PCR grade water substituted swab sample. Pink colour indicates negative result, yellow signals positive test result.

## Data Availability

All data generated or analysed during this study are included in this published article and its supplementary information files.

## Abbreviations

*Bst*: *Bacillus stearothermophilus*
CDC: Center of Disease Control and Prevention
COVID-19: Corona virus disease 2019
CPE: Cytopathic effect
Cq: Cycle of quantification
ddPCR: Droplet digital PCR
DMEM: Dibco modified Eagles media
IC: Internal control
LAMP: Loop-mediated isothermal amplification
LGL: Bavarian Health and Food Safety Authority
LOD: Limit of detection
MERS-CoV: Middle East respiratory syndrome coronavirus
NTC: Non-template control
PCR: Polymerase chain reaction
POCT: Point-of-care testing
qPCR: Real-time polymerase chain reaction
RSV: Respiratory syncytial virus
RT: Reverse transcription
SARS-CoV: Severe acute respiratory syndrome coronavirus
SARS-CoV-2: Severe acute respiratory syndrome coronavirus 2
