# Supplementary material for: Rapid point-of-care detection of SARS-CoV-2 using reverse transcription loop-mediated isothermal amplification (RT-LAMP)
Source: Virol J. 2020 Oct 21;17:160. doi: 10.1186/s12985-020-01435-6 (PMC7576985; doi:10.1186/s12985-020-01435-6)
Supplement: Supplementary file 1 — Additional file 1: Figure S1. Positions of LAMP primers in SARS-CoV-2 genome. LAMP primers of genes ORF8 (A) and N (B) aligned to the SARS-CoV-2 reference sequence. Arrowheads indicate orientation of the primer. Primers F1c and F2 together build the FIP primer, whereas primers B1c and B2 together build the BIP primer of the different LAMP primer sets. [file 12985_2020_1435_MOESM1_ESM.pdf]

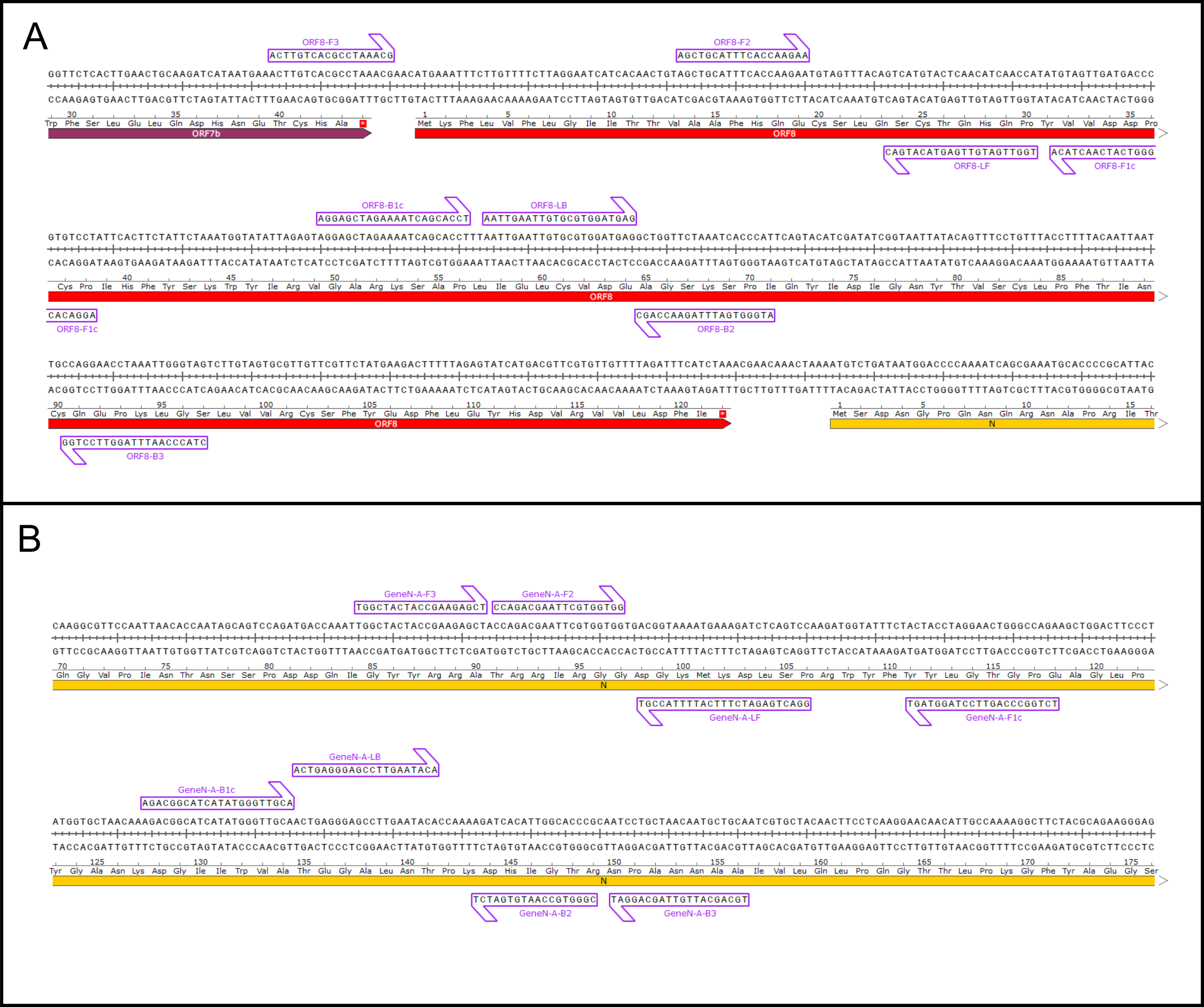

A
ORF8-F3
ACTTGTCACGCCTAAACG
ORF8-F2
AGCTGCATTTCACCAAGAA
ORF7b
ORF8
CAGTACATGAGTTGTAGTTGGT
ORF8-LF
ACATCAACTACTGGG
ORF8-F1c
ORF8-B1c
AGGAGCTAGAAAATCAGCACCT
ORF8-LB
AATTGAATTGTGCGTGGATGAG
CACAGGA
ORF8-F1c
CGACCAAGATTTAGTGGGTA
ORF8-B2
GGTCCTTGGATTTAACCCATC
ORF8-B3
N
B
GeneN-A-F3
TGGCTACTACCGAAGAGCT
GeneN-A-F2
CCAGACGAATTCGTGGTGG
N
TGCCATTTTACTTTCTAGAGTCAGG
GeneN-A-LF
TGATGGATCCTTGACCCGGTCT
GeneN-A-F1c
GeneN-A-LB
ACTGAGGGAGCCTTGAATACA
GeneN-A-B1c
AGACGGCATCATATGGGTTGCA
TCTAGTGTAACCGTGGGC
GeneN-A-B2
TAGGACGATTGTTACGACGT
GeneN-A-B3
