# Supplementary material for: Rapid point-of-care detection of SARS-CoV-2 using reverse transcription loop-mediated isothermal amplification (RT-LAMP)
Source: Virol J. 2020 Oct 21;17:160. doi: 10.1186/s12985-020-01435-6 (PMC7576985; doi:10.1186/s12985-020-01435-6)
Supplement: Supplementary file 2 — Additional file 2: Table S1. Seven different swab types for suitability test of different swab types for RT-LAMP. [file 12985_2020_1435_MOESM2_ESM.docx]

Table S1:

| Name in study | Product labelling | Order number | Picture |
| --- | --- | --- | --- |
| Swab 1 | ∑ Transwab | #MW176S, MWE | 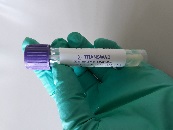 |
| Swab 2 | eSWAB | #480, Copan | 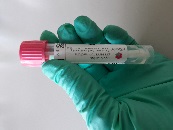 |
| Swab 3 | ∑ Virocult | #MW950S, MWE | 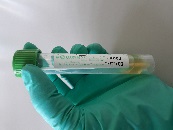 |
| Swab 4 | Virus Sampling Kit | #MT0301, Yocon | 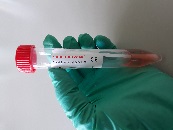 |
| Swab 5 | Liquid Amies Midia – virus transport kit | Jinan Babio Biotechnology | 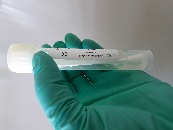 |
| Swab 6 | Virus Sample Stabilizer | #R513-02, Vazyme | 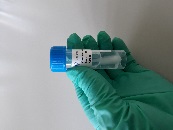 |
| Swab 7 | Dry Swab | #155C, Copan | 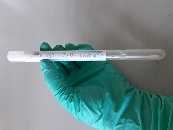 |
