# Supplementary figures and images for: Rapid point-of-care detection of SARS-CoV-2 using reverse transcription loop-mediated isothermal amplification (RT-LAMP)
Source: Virol J. 2020 Oct 21;17:160. doi: 10.1186/s12985-020-01435-6 (PMC7576985; doi:10.1186/s12985-020-01435-6)

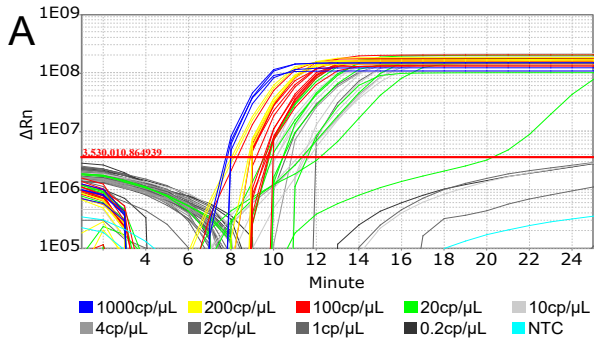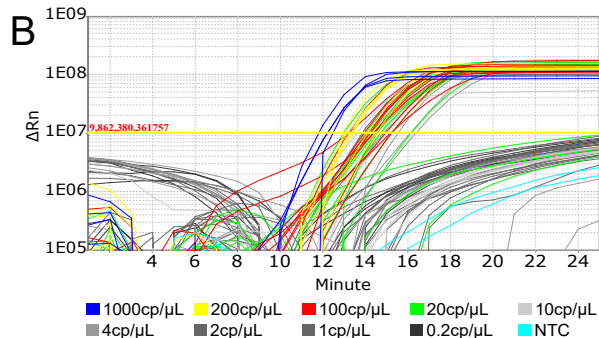

Supplement: Supplementary file 3 — Additional file 3: Figure S2. Determination of the sensitivity of the assay. Isolated RNA from SARS-CoV-2 infected Vero cell culture supernatant was quantified using RT-ddPCR and serially diluted to determine the LOD. 1000 copies/µL and 200 copies/µL dilutions were analysed in six replicates, while every lower dilution (1000–0.2 copies/µL) were analysed in 12 replicates. For both target genes ORF8 (A) and N (B) the LOD is 100 copies/µL as the last dilution where all 12 replicates are positive. For non-template control (NTC) PCR grade water substituted SARS-CoV-2 RNA. [file 12985_2020_1435_MOESM3_ESM.pdf]

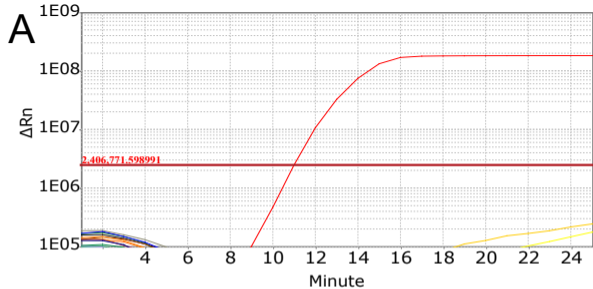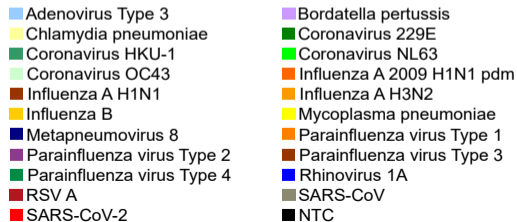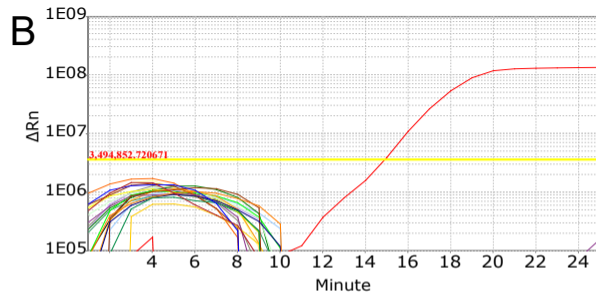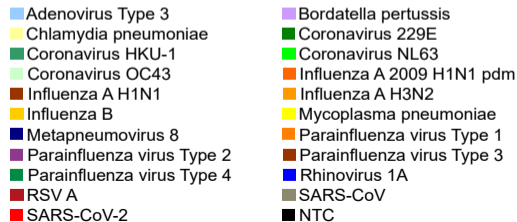

Supplement: Supplementary file 4 — Additional file 4: Figure S3. Specificity of the assay. SARS-CoV Frankfurt 1 RNA and nucleic acid extracts from 20 different samples from respiratory pathogens were tested with RT-LAMP targeting ORF8 (A) and N (B) to determine cross reactivity. For non-template control (NTC) PCR grade water was used. [file 12985_2020_1435_MOESM4_ESM.pdf]

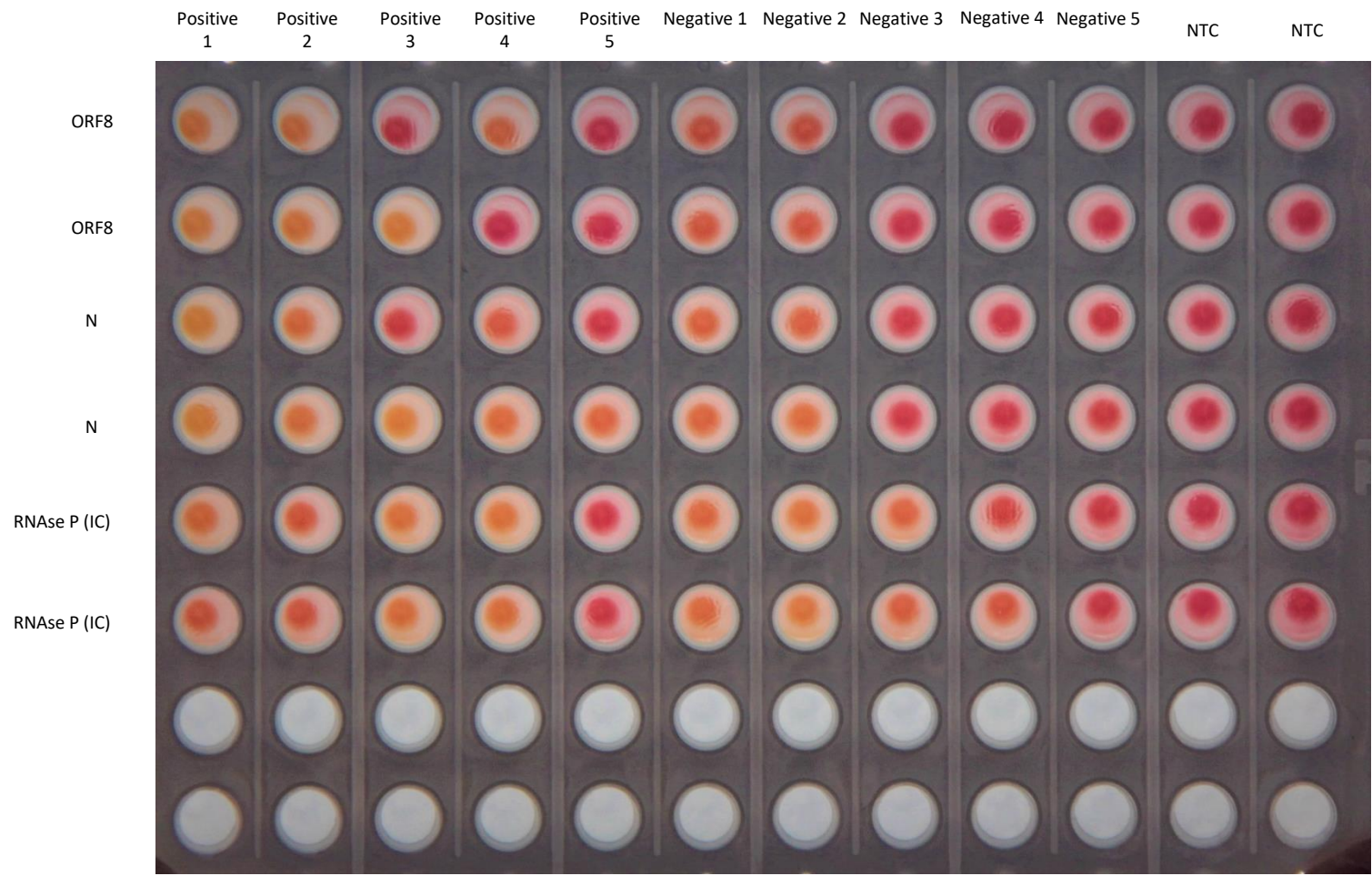

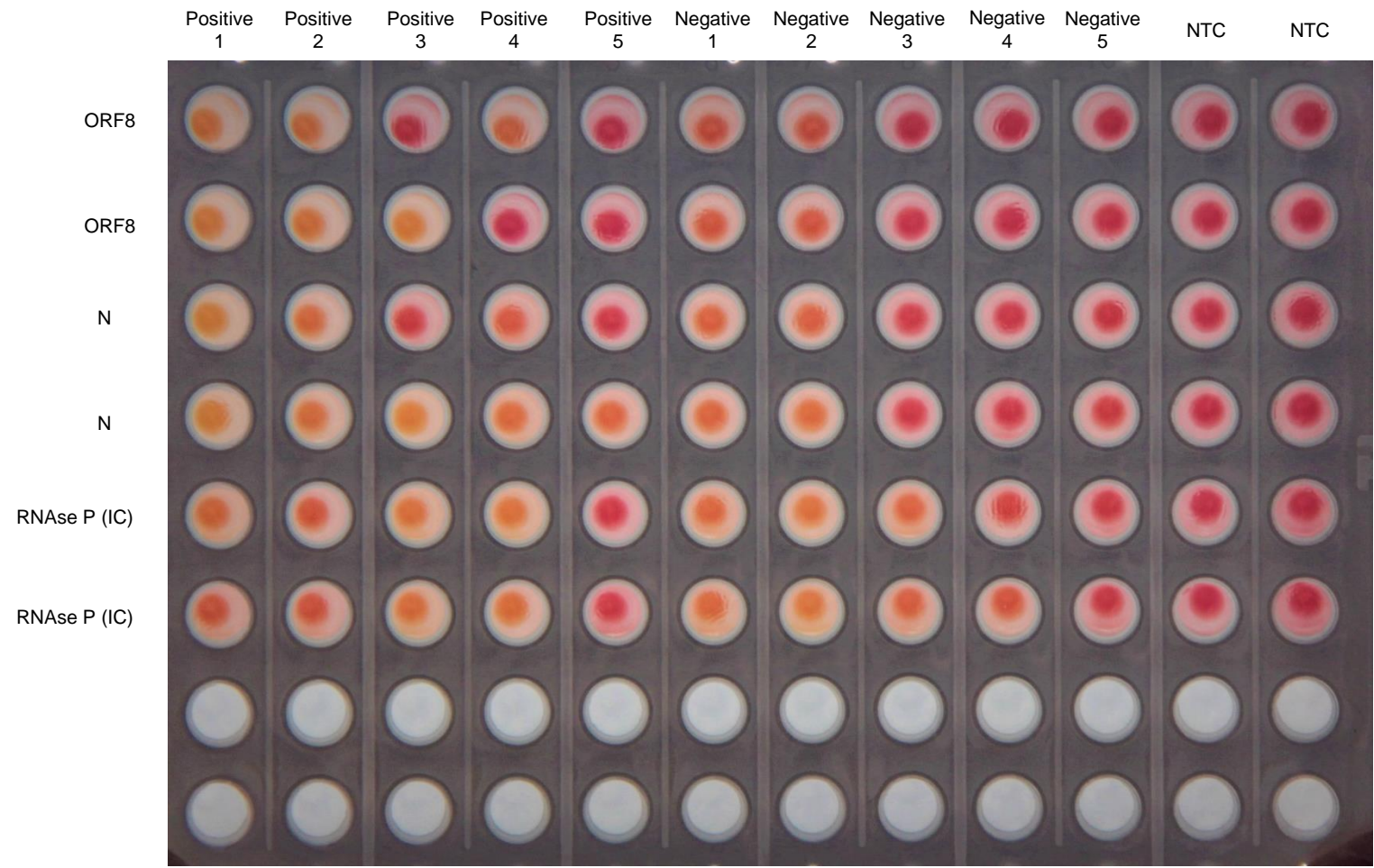

Supplement: Supplementary file 5 — Additional file 5: Figure S4. Colorimetric read-out of SARS-CoV-2 RT-LAMP. Positive samples 1–5 and Negative samples 1–5 were pre-heated at 90 °C for 5 min before being directly pipetted into RT-LAMP for SARS-CoV-2 targeting ORF8 and N. As internal control (IC) RNase P was additionally targeted. WarmStart Colorimetric LAMP 2x Master Mix (#M1800S, NEB, Ipswich, USA) was used by the manufacturer’s instructions with 5 µL of heated swab sample. For non-template control (NTC) PCR grade water substituted swab sample. Pink colour indicates negative result, yellow signals positive test result. [file 12985_2020_1435_MOESM5_ESM.pdf]
